# Cryoglobulinemic membranoproliferative glomerulonephritis associated with mucosa-associated lymphoid tissue lymphoma treated with rituximab 

**DOI:** 10.5414/CNCS108740

**Published:** 2016-01-19

**Authors:** Austin Y. Ha, Nicole Noronha, Patrick Gleason, Jonathan N. Winkler, James N. Butera, Kammi J. Henriksen, Susie L. Hu

**Affiliations:** 1 Warren Alpert Medical School of Brown University,; 2 Department of Medicine,; 3 Division of Hematology-Oncology, Department of Medicine, Warren Alpert Medical School of Brown University, Rhode Island Hospital, Providence, RI,; 4 Department of Pathology, University of Chicago Medicine, Chicago, IL, and; 5 Division of Kidney Disease and Hypertension, Department of Medicine, Warren Alpert Medical School of Brown University, Rhode Island Hospital, Providence, RI, USA

**Keywords:** cryoglobulinemia, membranoproliferative glomerulonephritis, mucosa-associated lymphoid tissue lymphoma, rituximab

## Abstract

Cryoglobulinemia and mucosa-associated lymphoid tissue (MALT) lymphoma are diseases characterized by B-cell dysregulation and overproduction of antibodies. Vasculitis and cutaneous manifestations are common, but renal involvement is rare. A 65-year-old woman with type 1 cryoglobulinemia and MALT lymphomas of the right lacrimal and parotid glands successfully treated by excision and chemoradiotherapy, presented with dyspnea on exertion, edema, and hematuria. Renal biopsy findings revealed type 1 cryoglobulinemic glomerulonephritis. She underwent treatment with high-dose oral prednisone and intravenous rituximab with subsequent return of creatinine to baseline levels. To our knowledge, this is the first report of a patient in whom type 1 cryoglobulinemia, multiple MALT lymphomas, and MPGN with IgM cryoglobulin deposits coexist. Evidence for rituximab is sparse with widely varying protocols and mixed results. There is a need for high quality evidence in the treatment of these conditions.

## Introduction 

B-cell dysregulation underlies non-Hodgkin mucosal-associated lymphoid tissue (MALT) B-cell lymphoma and cryoglobulinemia. Monoclonal IgM paraprotein elevation, as seen in type I cryoglobulinemia, is most commonly observed in non-Hodgkin lymphomas (NHL) [1]. Systemic vasculitis may be the first presenting symptom of MALT B-cell lymphomas [2]. Glomerular involvement in NHL is rare, but when it occurs, membranoproliferative glomerulonephritis (MPGN) is the most common lesion [3, 4]. 

MALT lymphoma of the lacrimal gland is also uncommon and usually characterized by a slowly enlarging orbital mass [5]. It has not previously been associated with cryoglobulinemia and its renal manifestations. 

To our knowledge, there is no report of MALT lymphoma of the lacrimal gland, MPGN, and cryoglobulinemia in one patient. We report a case of a patient with a history of systemic type I cryoglobulinemic vasculitis and MALT lymphoma of the lacrimal and parotid glands presenting with MPGN. Additionally, we discuss her clinical course and response to treatment with rituximab. 

## Case history 

A 65-year-old woman presented to the Rhode Island Hospital Emergency Department with 10 days of dyspnea on exertion, edema and hematuria. Seven years prior, she was diagnosed with type 1 cryoglobulinemia (monoclonal IgM with κ-light chains) in the setting of a purpuric rash. Two years later, she developed MALT lymphoma of the right lacrimal gland requiring excision and radiotherapy. Subsequently, MALT lymphoma of the right parotid gland and associated peri-portal and hilar lymphadenopathy were successfully treated with excision and chemotherapy with cyclophosphamide, prednisone, rituximab, and vincristine. She was also prescribed lisinopril, amlodipine, metoprolol and furosemide for hypertension and glipizide for diabetes mellitus. 

Physical examination on presentation was notable for tachycardia and 1+ bilateral pedal edema without cyanosis or clubbing. Blood pressure was 146/84 on her antihypertensive regimen. Laboratory tests were remarkable for blood urea nitrogen of 44 mg/dL, peak creatinine of 2.4 mg/dL (with a baseline of 1.02 mg/dL 5 months prior), nadir albumin of 2.8 g/dL, total cholesterol of 161 mg/dL, white blood cell count of 10,900/uL, and urinalysis with 177 red blood cells (some dysmorphic) per high-power field. Urine cytology showed no evidence of malignancy. 

Peak urine protein-to-creatinine ratio was 7.35 g/g. Complements were low: C3 was 65.6 mg/dL, C4 < 1.7 mg/dL and CH50 < 10 mg/dL. Rheumatoid factor was elevated at 285 IU/mL. Anti-neutrophil cytoplasmic antibody (ANCA) and anti-nuclear antibody (ANA) screens were negative. Cryocrit was positive at 158 µL/mL serum, and serum immunofixation electrophoresis revealed monoclonal IgM with κ-light chains. Hepatitis B, hepatitis C, and HIV serologies were negative. No other significant laboratory abnormalities (electrolytes, uric acid, glucose) were present. Chest X-ray revealed bilateral pleural effusions, which were removed with thoracentesis. 

**Figure 1.**
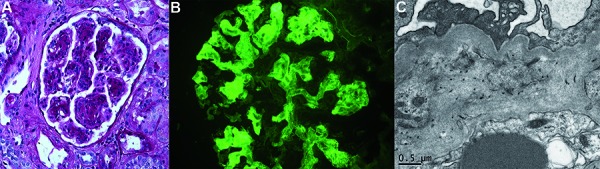
A: The sampled glomeruli showed membranoproliferative features with accentuated lobulation, mesangial and frequent endocapillary hypercellularity, and duplication of the glomerular basement membranes (PAS, 400×); B: Direct immunofluorescence revealed strong granular mesangial and frequent capillary wall staining for IgM, C3, and κ-light chains only (κ-light chain shown, 400×); C: Electron microscopy showed numerous immune-type electron dense deposits in mesangial and subepithelial to intramembranous locations, which focally demonstrated a fibrillary to microtubular substructure (19,000×).

Renal biopsy performed 2 weeks later for persistently elevated creatinine revealed glomerulonephritis with a membranoproliferative pattern of injury, and several glomeruli contained small cellular to fibrocellular crescents (Figure 1A). Hyaline pseudothrombi were not identified in the glomerular capillaries. A Congo red stain was negative for amyloid deposition. By direct immunofluorescence, there was granular mesangial and capillary wall staining for IgM (3+), C3 (2-3+), and κ-light chain only (3-4+) (Figure 1B). Ultrastructural evaluation revealed numerous mesangial and subendothelial deposits with a fibrillary to microtubular substructure (Figure 1C). Taken together, the clinicopathologic findings were most consistent with a type 1 cryoglobulinemic glomerulonephritis. She was treated with oral prednisone 60 mg daily and intravenous rituximab (two 1 g doses separated by 2 weeks) with subsequent decrease in creatinine to 1.4 mg/dL and urine protein to creatinine ratio to 0.4 g/g. Lisinopril was discontinued at the time of her creatinine elevation. 

**Figure 2.**
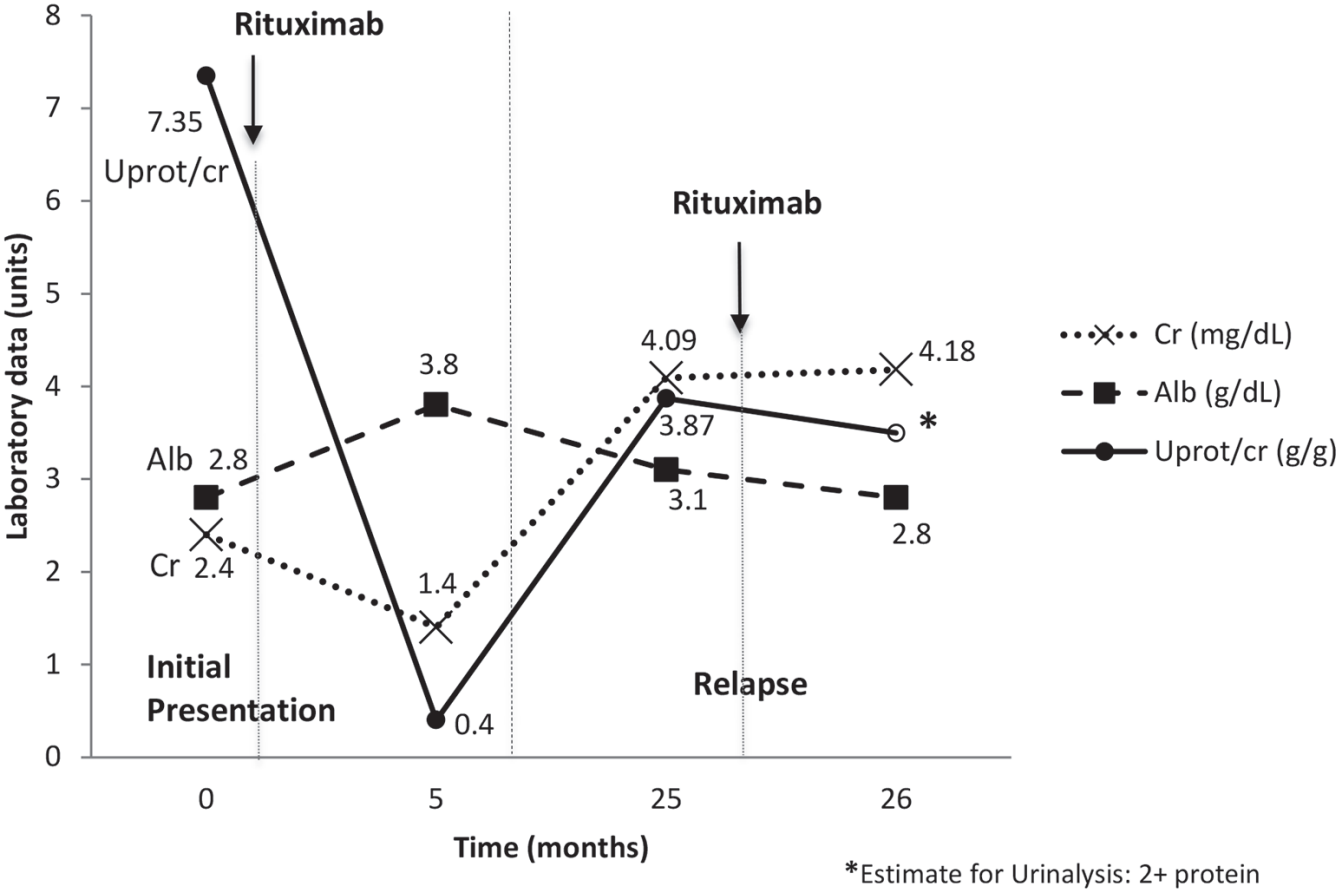
Clinical course with rituximab treatment. Alb = albumin; Cr = creatinine; Uprot/cr = urine protein-to-creatinine ratio.

Three years later, the patient had recurrent acute kidney injury due to MPGN requiring rituximab therapy resulting in minimal improvement in symptoms or laboratory values. Given persistent symptoms of volume overload and clinical decline, she was initiated on hemodialysis 3 months later. Figure 2 summarizes the trends in relevant laboratory values over time. 

## Discussion 

Type I cryoglobulinemia is the least common type of cryoglobulinemia. Monoclonal IgM elevation, in particular, is strongly associated with underlying lymphoproliferative disorders including Waldenstrom’s macroglobulinemia, multiple myeloma, MALT lymphoma or monoclonal gammopathy of unknown significance [6]. MALT lymphoma is a subtype of NHL characterized by B-cell expansion, which may result in increased antibody production. Monoclonal cryoglobulinemia can be the first manifestation of MALT lymphoma, and NHL is estimated to be up to 35 times more likely in patients with cryoglobulinemia [2, 7]. In type I cryoglobulinemia, the overproduction of monoclonal antibodies can damage small vessels and tissues through direct deposition, and can also induce hyperviscosity of the blood [7]. Kidney involvement in MALT lymphoma is unusual, but the most commonly reported glomerular lesion is MPGN most often secondary to light chain monoclonal antibody deposition, although neoplastic disease and thrombotic microangiopathy have also been reported [3, 4]. One case series by Neel et al. [8], showed renal involvement in ~ 32% of cases, where IgG deposition was more common in those with MPGN. Cryoglobulin levels did not correspond to disease severity. 

Rituximab is a murine monoclonal antibody that targets the transmembrane protein CD20 found in developing and mature B-cells and originally designed for treatment of NHLs. Rituximab has been used with moderate success in treatment of type II and type III cryoglobulinemia [9]. A retrospective chart review by Bryce et al. [10] suggests that rituximab was most effective for cutaneous manifestations of type II cryoglobulinemia and minimally effective for renal disease. However, the majority of case reports cited an improvement of cryoglobulinemic nephropathy after rituximab treatment [11]. 

The mainstay of treatment for type I cryoglobulinemia is treatment of the underlying disease process, most often a lymphoproliferative disease. Our patient had achieved complete remission of her MALT lymphomas with chemotherapy including cyclophosphamide, prednisone, rituximab, and vincristine. Rituximab is a mechanistically promising therapy for type I cryoglobulinemia associated with MALT lymphoma because it specifically targets B-cells which overproduce IgM cryoglobulins resulting in kidney damage. Response to rituximab therapy have been mixed. Akiyama et al. [12] reported improvement in renal function and resolution of nephrotic syndrome in a woman with MPGN and IgM-κ cryoglobulin elevation who was treated with a combination of rituximab, prednisolone, and plasma exchange. Similarly, Pandrangi et al. [13] found that rituximab treatment was effective in treating a patient presenting with type I cryoglobulinemia-associated MPGN with an increased number of CD20+ B cells. A case series of 6 patients by Dillon et al. [14] demonstrated decreased proteinuria with rituximab in patients with type I MPGN, but similar improvements in creatinine levels were not seen. Finally, a review by Harel et al. [9] suggests that rituximab is more effective in treating kidney disease among patients with IgM monoclonal elevation but is of limited value in patients with IgG elevation. 

In this report, we describe a patient with a history of MALT lymphoma presenting with monoclonal IgM cryoglobulinemia-associated membranoproliferative glomerulonephritis. Initial treatment with high-dose corticosteroids and rituximab resulted in improvement in renal function; however, there was no response to rituximab after relapse, possibly due to irreversible sclerosis. To our knowledge, no report describes these three rare associations coexisting in one patient. There is limited evidence supporting rituximab for the treatment of cryoglubulinemia-associated MPGN, and reports vary widely in their treatment protocols. Our case highlights an interesting association and the need and challenges for evidence-based approach in the treatment of these rare conditions. 

## Conflict of interest 

No support, financial or otherwise was obtained for this work.
